# Do entrapment, injuries, outcomes and potential for self-extrication vary with age? A pre-specified analysis of the UK trauma registry (TARN)

**DOI:** 10.1186/s13049-021-00989-w

**Published:** 2022-03-05

**Authors:** Tim Nutbeam, Anthony Kehoe, Rob Fenwick, Jason Smith, Omar Bouamra, Lee Wallis, Willem Stassen

**Affiliations:** 1 Emergency Department, University Hospitals Plymouth NHS Trust, Plymouth, UK; 2 Devon Air Ambulance Trust, Exeter, UK; 3 Division of Emergency Medicine, University of Cape Town, Cape Town, South Africa; 4 Emergency Department, Somerset NHS Foundation Trust, Taunton, UK; 5 University Hospitals Birmingham, Birmingham, UK; 6 Academic Department of Military Emergency Medicine, Royal Centre for Defence Medicine, Birmingham, UK; 7 Trauma Audit Research Network, University of Manchester, Manchester, UK

**Keywords:** Older patients, Extrication, Accidents, Traffic, Spinal injury, Emergency Medical Services

## Abstract

**Background:**

Motor vehicle collisions (MVCs), particularly those associated with entrapment, are a common cause of major trauma. Current extrication methods are focused on spinal movement minimisation and mitigation, but for many patients self-extrication may be an appropriate alternative. Older drivers and passengers are increasingly injured in MVCs and may be at an increased risk of entrapment and its deleterious effects. The aim of this study is to describe the injuries, trapped status, outcomes, and potential for self-extrication for patients following an MVC across a range of age groups.

**Methods:**

This is a retrospective study using the Trauma Audit and Research Network (TARN) database. Patients were included if they were admitted to an English hospital following an MVC from 2012 to 2019. Patients were excluded when their outcomes were not known or if they were secondary transfers. Simple descriptive analysis was used across the age groups: 16–59, 60–69, 70–79 and 80+  years. Logistic regression was performed to develop a model with known confounders, considering the odds of death by age group, and examining any interaction between age and trapped status with mortality.

**Results:**

70,027 patients met the inclusion criteria. Older patients were more likely to be trapped and to die following an MVC (*p* < 0.0001). Head, abdominal and limb injuries were more common in the young with thoracic and spinal injuries being more common in older patients (all *p* < 0.0001). No statistical difference was found between the age groups in relation to ability to self-extricate. After adjustment for confounders, the 80 + age group were more likely to die if they were trapped; adjusted OR trapped 30.2 (19.8–46), not trapped 24.2 (20.1–29.2).

**Conclusions:**

Patients over the age of 80 are more likely to die when trapped following an MVC. Self-extrication should be considered the primary route of egress for patients of all ages unless it is clearly impracticable or unachievable. For those patients who cannot self-extricate, a minimally invasive extrication approach should be employed to minimise entrapment time.

## Background

Motor vehicle collisions (MVCs) are a frequent cause of trauma and death for patients of all ages [1]. Following an MVC some patients will be trapped [1]. Only around 10% of such patients are physically trapped by deformation of the vehicle, requiring dismantling of the vehicle and space creation by rescue services [2]. The overwhelming majority are trapped due to painful injuries inhibiting movement or physiological impairment rendering voluntary movement difficult [3]. However, often it is rescuer or casualty concerns about exacerbating secondary spinal injury which prevent self or minimally assisted extrication [4, 5].

Being trapped through any of these mechanisms is associated with excess mortality [1]. It is not yet clear whether this can be mitigated by reducing extrication time [6]. Extrication strategies have historically focused on movement mitigation such as roof removal techniques, which inherently take longer to deliver than self-extrication. However, recent work has demonstrated the biomechanical and time advantages of self-extrication over tool-based techniques [7]. Minimising entrapment time reduces avoidable delays to diagnosis and clinical interventions, whilst also reducing the detrimental effects of environmental exposure. A combination of these factors and others may lead to the excess mortality seen in trapped casualties.

Average life expectancy is increasing throughout most of the world, with the most rapidly growing segment of the population being people aged over 60 years [8]. With this changing demographic, healthcare systems have witnessed a disproportionate rise in older patients suffering from major trauma, with this group now representing over 50% of the major trauma cases reported in the UK [8, 9]. There are more older drivers and passengers on the roads than ever before, with older road users representing 12% of car driving license holders and 9% of road miles travelled [10, 11]. Older patients have a higher mortality rate, with those over 70 representing a disproportionate 20% of all car driver deaths [12].

Older casualties may be at increased risk of entrapment through decreased baseline mobility, a propensity to frailty and vulnerability to certain types of injury [8]. It is unknown if older patients are more at risk from the dangerous effects of prolonged entrapment. Extrication may be delayed due to rescuer perceptions about the incidence of spinal injury in this group and their ability to self-extricate [13].

The aims of this study were to describe the rate of entrapment, the type and frequency of injuries, and outcomes in different age groups, and whether there is disproportionate mortality from entrapment in older patients. We also compared the incidence of factors likely to impede self-extrication between the groups.

## Methods

This is a retrospective review of the UK Trauma Audit and Research Network (TARN) database. TARN is the UK national trauma registry into which all Major Trauma Centres submit data on severely injured patients. TARN moved from voluntary to mandatory submission of data from MVCs in 2012. Eligibility criteria include trauma patients who are admitted to hospital for ≥ 72 h, are admitted to a critical care unit, die in hospital, or are transferred to another hospital for specialist trauma care. Isolated closed fractures of the limbs and hip fractures in patients over 65 are excluded. TARN includes patient demographics, initial physiology, treatment interventions, detail of injuries and in some circumstances (including MVCs) their trapped status.

This study describes the rate of entrapment by age group, considering the effect of being trapped on outcomes and whether this effect modifies with age. Reporting the rate and type of spinal injuries, other severe potentially time critical injuries and traumatic and physiological challenges to self-extrication by age group will inform choice of extrication strategy [14, 15].

Patients were included if they were admitted between January 2012 and December 2019, were involved in an MVC, were admitted directly to an English hospital, and had a known outcome. Patients were excluded when their trapped status was not known. For patients who met the inclusion criteria, data fields including age, trapped status, injury severity score (ISS), abbreviated injury score (AIS) for each body region were reported. In addition we report details of spinal injury and other severe injuries that we have previously defined [2].

Adults were categorised into age groups: 16–59, 60–69, 70–79 and 80+ years. These age groups were selected as they have previously been defined by TARN [8]. The 80+ age group were considered as a whole to prevent the statistical artifact associated with small sample sizes. Simple descriptive analysis was used to define the characteristics of the groups by age category and trapped status. A two-tailed t-test was used to compare means and Mann–Whitney U test for comparing medians. The Chi square test for uniform distribution was used for categorical variables. *P* values of less than 0.01 were considered significant due to multiple analyses being performed. Logistic regression was used to develop a model with the following known confounders: sex, ISS, GCS, Charlson comorbidity index and entrapment status as exposure variables, considering the odds of death by age group, and examining any interaction between age and trapped status with mortality. Missing values for GCS were imputed under the assumption of a mechanism of missing at random (MAR). SPSS (IBM Corp v.23 Armonk, NY), Stata (StataCorp. 2015. Stata Statistical Software: Release 14. College Station, TX) and R (Integrated Development for R. RStudio, PBC, Boston, MA, v.1.4) software were used for the analyses.

A literature review failed to identify previous studies or guidance which indicates which patients are suitable for self-extrication. All parameters available through the TARN data set were considered by the research group; factors were identified which the group from their clinical and operational experience considered likely to affect the ability of a patient to successfully self-extricate. Factors where consensus was achieved were GCS 12 or less; Spine, Limb or Pelvis Abbreviated Injury Scale (AIS) score of 3+; or a systolic blood pressure of < 90 mmHg. Patients where none of these factors were present were considered as having a high potential for self-extrication.

TARN data analyses are conducted using anonymised data which is governed by a code of practice approved by the Confidentiality Advisory Group who are appointed by the Health Research Authority. Additional individual ethical approval was not required for this analysis.

## Results

**Fig. 1:**
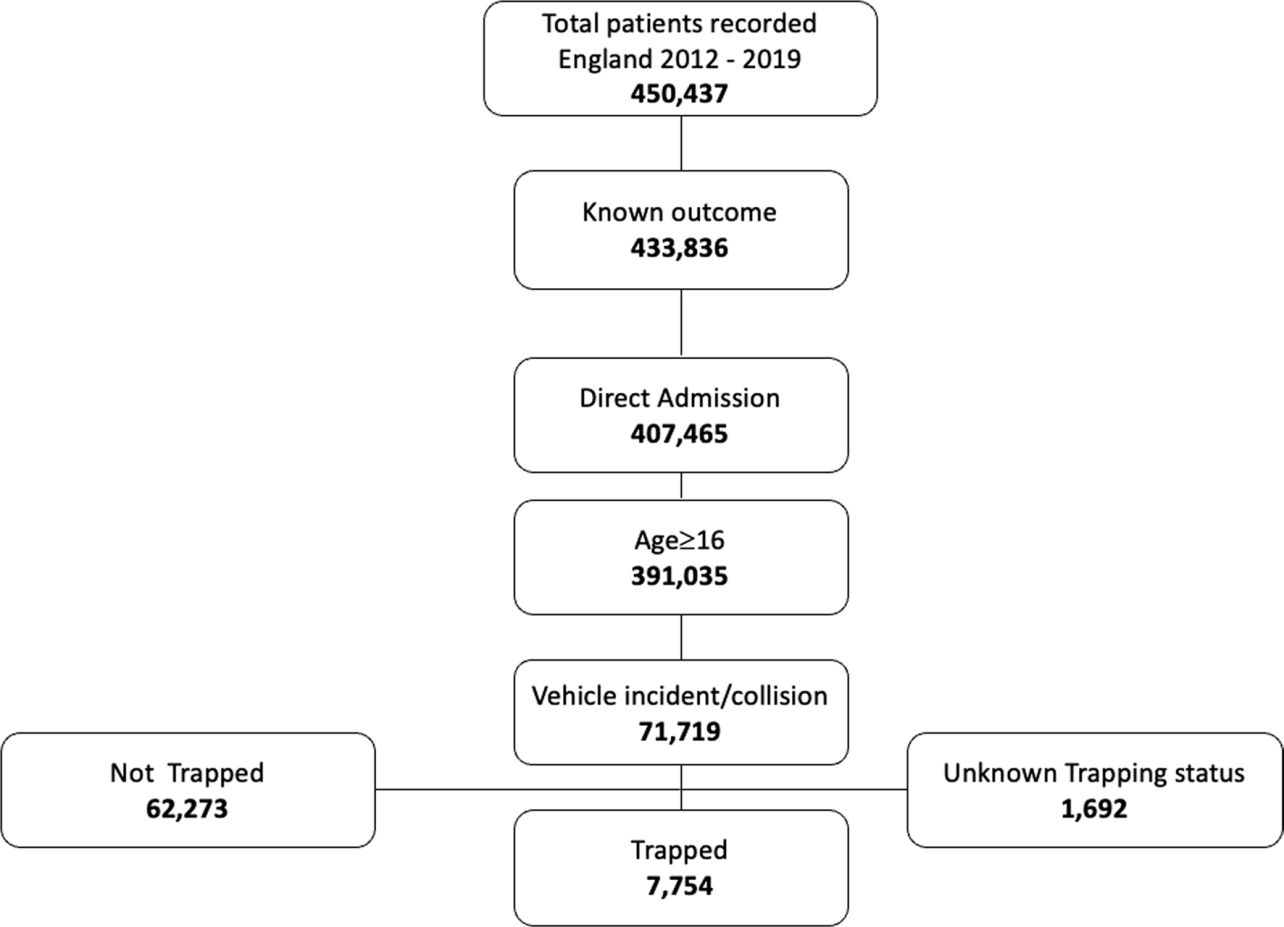
STROBE diagram

Between 2012 and 2019 there were 450,437 major trauma cases identified on the TARN database of whom 70,027 met the inclusion criteria (Fig. 1).

The characteristics of each group are summarized in Table 1. The systolic blood pressure increased with age, whilst the pulse, respiratory rate, oxygen saturations and GCS demonstrate statistical though not clinical differences between the groups with no age-dependent trend. With large data-sets there is a well reported tendency for the identification of statistically significant but clinically inconsequential effects [16].

**Table 1:** Demographics and mortality by age

Age groups	Total	16–59	60–69	70–79	80+
Total number of cases	70,027	51,868	7605	5733	4821
Male, n (%)	51,852 (74%)	40,957 (79%)	5232 (68.8%)	3197 (55.8%)	2466 (51.2%)
ISS, median (IQR)	13 (9–22)	13 (9–24)	13 (9–22)	13 (9–24)	13 (9–22)
Systolic BP, mean (SD)	133 (28)	129 (25)	140 (30)	145 (33)	149 (34)
Pulse rate, mean (SD)	87 (22)	88 (22)	83 (21)	83 (22)	83 (21)
Respiratory rate, mean (SD)	20 (7)	20 (7)	20 (7)	20 (7)	20 (7)
Oxygen saturation, mean (SD)	96 (8)	96 (8)	96 (8)	95 (9)	95 (7)
GCS, median (IQR)	15 (15–15)	15 (15–15)	15 (15–15)	15 (15–15)	15 (15–15)
Trapped, n (%)	7754 (11.1%)	5642(10.9%)	807 (10.6%)	756 (13.2%)	549 (11.4%)
Mortality, n (%)	3868 (5.5%)	2125 (4.1%)	391 (5.1%)	564 (9.8%)	788 (16.4%)

IQR interquartile range, SD standard deviation

Statistically significant differences (*p* < 0.0001) were found across all groups apart from in Respiratory rate and GCS catergories

The median ISS was similar across the age groups. Thirty-day mortality increased with increasing age from 4.1% (16–59) to 16.4% (80+).

**Fig. 2:**
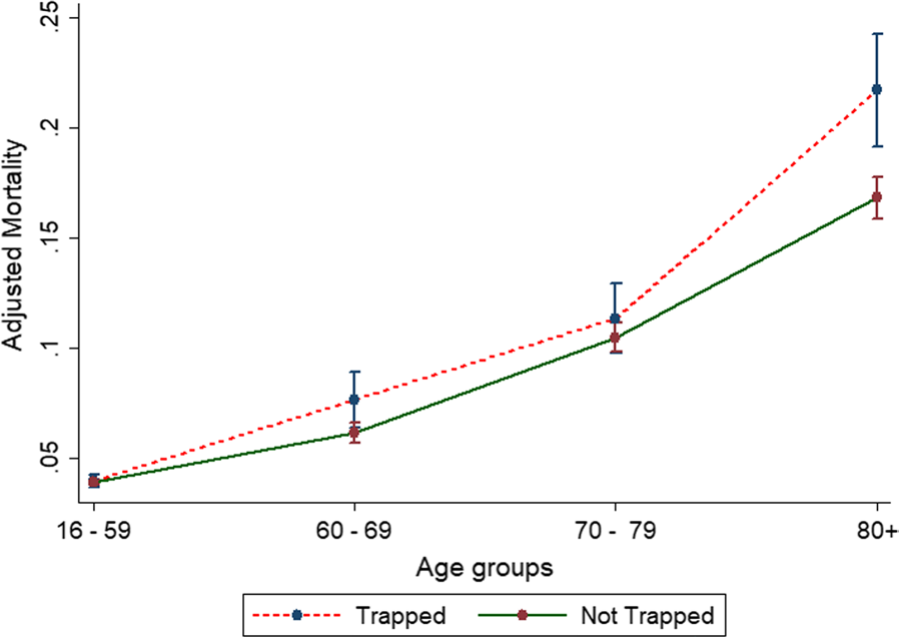
Age and adjusted odds of death

**Fig. 3:**
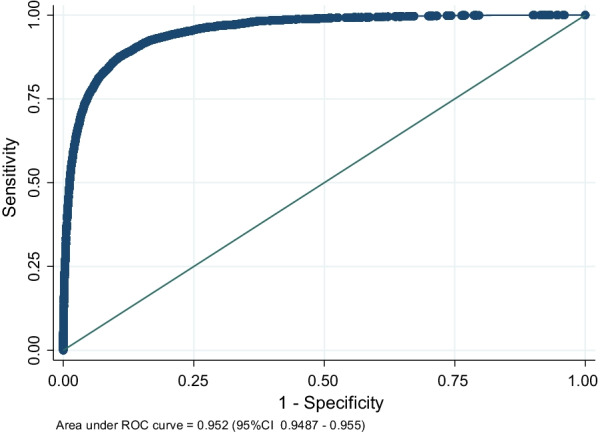
Receiver operator curve for model (*Gender, ISS, GCS, Charlson comorbidity index and entrapment status as exposure variables)

As shown in Table 2, unadjusted and adjusted odds of death increased with age. Trapped patients over 80 had an increased mortality rate compared to those that were not trapped (Fig. 2). This model performed well, with a discrimination area under the receiver operator curve (ROC, C-statistic) of 0.952 (95% CI 0.948–0.955) as shown in Fig. 3.

**Table 2:** Trapped status and mortality by age

Age groups	Trapped at scene				Not trapped at scene			
	16–59	60–69	70–79	80+	16–59	60–69	70–79	80+
Unadjusted odds ratio of death (95% CI)	1	1.1 (0.9–1.5)	1.7 (1.4–2.2)	4.4 (3.6–5.5)	1	1.3 (1.2–1.5)	2.7 (2.5–3.0)	4.6 (4.2–5.1)
Adjusted odds ratio of death (95% CI)	1	3.7 (2.3–5.9)	8.5 (5.5–13.3)	30.2 (19.8–46)	1	2.8 (2.3–3.4)	8.7 (7.2–10.6)	24.2 (20.1–29.2)

Adjusted for gender, ISS, GCS, Comorbidity

In patients who were trapped, severe injuries occurred with similar frequency across all age categories (Table 3). Injuries (AIS 3+) to the head, face, abdomen and limbs were more common in the young (16–59, Table 4). Thoracic injuries were more frequent in those aged 60 or above.

**Table 3:** Severe and spinal injuries by age for trapped casualties

Injury	Total	Age group				Significance (*p*)
		16–59	60–69	70–79	80+	
Pelvic Ring Blood loss > 20%	71 (0.9%)	53 (0.9%)	9 (1.1%)	5 (0.7%)	4 (0.7%)	0.7578
Blood Loss > 20%	275 (3.5%)	210 (3.7%)	28 (3.5%)	20 (2.6%)	17 (3.1%)	0.4503
Tension pneumothorax	118 (1.5%)	91 (1.6%)	12 (1.5%)	8 (1.1%)	7 (1.3%)	0.6535
Spine multiple fractures	1078 (13.9%)	734 (13%)	114 (14.1%)	150 (19.8%)	80 (14.6%)	< 0.0001
Spine dens fracture	164 (2.1%)	87 (1.5%)	19 (2.4%)	37 (4.9%)	21 (3.8%)	< 0.0001
Spine compression grade 2 and 3	141 (1.8%)	98 (1.7%)	13 (1.6%)	17 (2.2%)	13 (2.4%)	0.5485
Spine unstable fracture	717 (9.2%)	502 (8.9%)	79 (9.8%)	94 (12.4%)	42 (7.7%)	0.0077
Spinal cord injury	526 (6.8%)	376 (6.7%)	51 (6.3%)	63 (8.3%)	36 (6.6%)	0.3452

**Table 4:** Injury site (AIS 3+) by age for trapped casualties

Anatomical site	Total	Age group				Significance (*p*)
		16–59	60–69	70–79	80+	
Head	1896 (24.5%)	1528 (27.1%)	135 (16.7%)	139 (18.4%)	94 (17.1%)	< 0.0001
Face	52 (0.7%)	43 (0.8%)	3 (0.4%)	5 (0.7%)	1 (0.2%)	< 0.0001
Thorax	4159 (53.6%)	2945 (52.2%)	438 (54.3%)	430 (56.9%)	346 (63%)	< 0.0001
Abdomen	950 (12.3%)	770 (13.6%)	74 (9.2%)	65 (8.6%)	41 (7.5%)	< 0.0001
Spine	844 (10.9%)	577 (10.2%)	96 (11.9%)	109 (14.4%)	62 (11.3%)	< 0.0001
Pelvic	895 (11.5%)	686 (12.2%)	82 (10.2%)	60 (7.9%)	67 (12.2%)	< 0.0001
Limb	2522 (32.5%)	2028 (35.9%)	232 (28.7%)	164 (21.7%)	98 (17.9%)	< 0.0001

The frequency of multiple spinal fractures, dens fractures, unstable fractures and cord injuries were highest in the 70–79 age group.

Statistically significant but not clinically significant differences were demonstrated across the physiological and injury-based considerations for self-extrication. The proportion of patients with injuries likely to preclude self-extrication was similar across the age groups (Table 5).

**Table 5:** Physiological and injury considerations for potential for self-extrication by age

Parameter	Total	Age group				Significance (*p*)
		16–59	60–69	70–79	80+	
Systolic BP < 90	418 (5.4%)	301 (5.3%)	48 (6.0%)	39 (5.2%)	30 (5.5%)	0.908
GCS 12 or less	1183 (15.3%)	1006 (17.8%)	68 (8.4%)	57 (7.5%)	52 (9.5%)	< 0.0001
Spine AIS3+	844 (10.9%)	577 (10.2%)	96 (11.9%)	109 (14.4%)	62 (11.3%)	< 0.0001
Pelvic AIS 3+	895 (11.5%)	686 (12.2%)	82 (10.2%)	60 (7.9%)	67 (12.2%)	< 0.0001
Limb AIS 3+	2522 (32.5%)	2028 (35.9%)	232 (28.7%)	164 (21.7%)	98 (17.9%)	< 0.0001
None of the above	3208 (41.4%)	2264 (40.1%)	343 (42.5%)	357 (47.2%)	244 (44.4%)	0.079

## Discussion

Patients over 80 years old are particularly vulnerable to the negative effects of entrapment following an MVC. Older patients are more likely to have chest and spinal injuries than younger patients—however, the overall rate of spinal injuries in comparison to other likely time dependent injuries remains low. Across the age groups, approximately 40% of patients who were trapped did not have injuries or physiological impairment likely to hinder self-extrication.

### Meaning of the study

This study offers fresh insights that are useful for those providing clinical care on scene, planning extrication strategies and supporting clinicians in enabling patients to self-extricate. Injuries of the head, thorax, face and limb are unlikely to benefit from a longer extrication strategy based on movement mitigation when other quicker routes such as self-extrication could be considered; these injuries may be time dependent and the extended time these patients remain in the vehicle will add to excess mortality related to bleeding and hypoxia [10, 17]. Gentle patient handling and movement mitigation may help with prevention of clot disruption in abdominal or pelvic injury [18], but these significant injuries often require blood product resuscitation and early access to hospital-based services for identification of injury (CT scan) and treatment (interventional radiology or damage control surgery) [19].

The small increased rate of spinal injuries in older patients may be because of the decreased bone density, muscle and ligament strength and degenerative changes causing narrowing of the spinal canal experienced by the older patient [13]. Recent work has identified that self-extrication results in less movement of the cervical and lumbar spine than other extrication types in healthy volunteers [7]. If these findings can be extrapolated to the injured population, self-extrication may present the best route of egress even for those with suspected spinal injuries.

Patients, and particularly older patients may have occult injuries [20]. As such, predicting a patient’s ability to self-extricate is complex. We suggest that self-extrication has significant advantages over more formal alternative extrication techniques and as such should be considered as a route of egress for all patients unless it is clearly impracticable or unachievable. The advantages of self-extrication for the patient include minimal entrapment time (self-extrication is quickest) and minimal movement [7]. For those patients who cannot self-extricate a minimally invasive extrication approach should be used—providing the patient with the necessary support to extricate from the vehicle with minimal cutting/space creation using the principles of gentle patient handling.

### Strengths and weaknesses

This is the largest analysis to date of trapped patients injured in motor vehicle collisions, which allows comparison of injury severity, injury type and outcomes for patients stratified by age.

TARN data may be incomplete, with patients aged over 60 having a lower level of data completeness than younger patients [8]. This study is based upon chronological age—the effects of which are subject to considerable variation between individuals [21]. This study does not specifically report frailty—which is likely to be an important factor both in a patient’s resilience to injury and their potential to recover successfully from injury and therefore affect both injury severity and mortality [22].

We have selected pragmatic physiological and injury-based criteria which are likely to affect the ability of a patient to participate in self-extrication. These criteria have not been validated in this setting but provide useful context.

This study is limited in that it does not report non-patient factors relating to the scene of a collision which will affect clinical decision making. We do not report type of vehicle, closing speed, vehicle damage or the use and/or deployment of restraint systems. Importantly we cannot distinguish between patients that are physically trapped and those that are medically trapped following their MVC.

### Unanswered questions and future research

Future work should focus on clearly defining patient groups that are not suitable for self-extrication. This may be through prospective data collection of extrication type and patient outcomes, expert consensus, and patient consultation. It is important to distinguish between patients who are physically trapped and those that are medically trapped, and this should be routinely collected on operational and medical data sets considering trapped patients post MVC.

## Conclusions

Patients over the age of 80 are more likely to die when trapped following an MVC. Older patients are more likely to have chest and spinal injuries than younger patients—however, the overall rate of spinal injuries remains low across all age groups. Older patients are no more likely to have injuries that would hinder self-extrication than younger patients.

Self-extrication should be considered the primary route of egress for patients of all ages apart from where it is clearly impracticable or unachievable. For those patients who cannot self-extricate a minimally invasive extrication approach should be employed to minimise entrapment time.

## Data Availability

The datasets used and/or analysed during the current study are available from the corresponding author on reasonable request.
